# Studies Regarding the Pharmaceutical Potential of Derivative Products from *Plantain*

**DOI:** 10.3390/plants11141827

**Published:** 2022-07-12

**Authors:** Marilena-Gabriela Olteanu Zaharie, Nicoleta Radu, Lucia Pirvu, Marinela Bostan, Mariana Voicescu, Mihaela Begea, Mariana Constantin, Catalina Voaides, Narcisa Babeanu, Viviana Roman

**Affiliations:** 1 Faculty of Biotechnology, University of Agronomic Sciences and Veterinary Medicine of Bucharest, 59 Marasti Boulevard, 011464 Bucharest, Romania; mzaharie@yahoo.com (M.-G.O.Z.); catalina.voaides@biotehnologii.usamv.ro (C.V.); narcisa.babeanu@biotehnologii.usamv.ro (N.B.); 2 Biotechnology Department, National Institute of Chemistry and Petrochemistry R & D of Bucharest, 202 Splaiul Independentei Street, 060021 Bucharest, Romania; marriconstantin@yahoo.com; 3 Biotechnology Department, National Institute of Chemical Pharmaceutical R & D, 112 Vitan Road, 031299 Bucharest, Romania; lucia.pirvu@yahoo.com; 4 Institute of Virology Stefan S. Nicolau, Center of Immunology, 285 Mihai Bravu Avenue, 030304 Bucharest, Romania; marinela.bostan@yahoo.com (M.B.); rviviana30@yahoo.com (V.R.); 5 Immunology Department, Victor Babes National Institute of Pathology, 050096 Bucharest, Romania; 6 Institute of Physical Chemistry Ilie Murgulescu, 202 Splaiul Independentei, 060021 Bucharest, Romania; voicescu@icf.ro; 7 Faculty of Biotechnical Systems Engineering, Politehnica University of Bucharest, 313 Splaiul Independentei, 060026 Bucharest, Romania; 8 Faculty of Pharmacy, University Titu Maiorescu, 178 Calea Vacaresti, 040051 Bucharest, Romania

**Keywords:** *Plantago lanceolata*, derivative products, antitumor activity, prebiotic activity

## Abstract

In this study, three types of extracts isolated from leaves of Plantain (*Plantago lanceolata*) were tested for their chemical content and biological activities. The three bioproducts are combinations of polysaccharides and polyphenols (flavonoids and iridoidic compounds), and they were tested for antioxidant, antifungal, antitumor, and prebiotic activity (particularly for polysaccharides fraction). Briefly, the iridoid-enriched fraction has revealed a pro-oxidant activity, while the flavonoid-enriched fraction had a high antioxidant potency; the polysaccharide fraction also indicated a pro-oxidant activity, explained by the co-presence of iridoid glycosides. All three bioproducts demonstrated moderate antifungal effects against *Aspergillus* sp., *Penicillium* sp., and dermatophytes, too. Studies in vitro proved inhibitory activity of the three fractions on the leukemic tumor cell line THP-1, the main mechanism being apoptosis stimulation, while the polysaccharide fraction indicated a clear prebiotic activity, in the concentration range between 1 and 1000 µg/mL, evaluated as higher than that of the reference products used, inulin and dextrose, respectively.

## 1. Introduction

*Plantain* (*Plantago lanceolata*) has been used since ancient times in traditional medicine, either as such or in the form of tinctures, decoctions, or infusions. At the base of the medicinal properties are the active principles [1,2,3,4,5], with antioxidant, and antitumor properties [6,7,8,9] as polysaccharides [10,11,12], polyphenols [3,5,8] alkaloids, terpenoids (ursolic acid, oleanolic acid), caffeic acid derivatives (plantamajoside, acteoside or verbascoside), iridoid glycosides (aucubin, catalpol), fatty acids (palmitic acid, linolenic acid, linoleic acid, myristic acid), vitamins [1,2,3], macroelements and microelements [13]. Analytical studies performed on the essential oil obtained by hydrodistillation from the leaves of *Plantain* showed that it contains a wide range of organic compounds, with fatty acids representing about 41% of the oils fraction [2]. According to the reported data, *Plantago lanceolata* contains up to 1 g of total phenolic compounds per 100 g of vegetal material, regardless of whether the plant is obtained from controlled crops or spontaneous flora [5,6,7]. The main phenolic compounds are caffeic acid and luteolin and apigenin derivates [5,7,8,11]. 

Caffeic acid derivatives found in *Plantaginaceaea* are the plantamajoside or acteoside type, the last one is also known as verbascoside [7]. The most prominent flavonoid derivates are luteolin 7-O-glucoside, hispidulin 7-O-glucuronide, luteolin 7-O-diglucoside, apigenin 7-O-glucoside, nepetin 7-O-glucoside, luteolin 6-hydroxy 4′-methoxy 7-galactoside, and homoplantaginin, baicalein, hispidulin, plantaginin, and scutallarein [9]. Studies also evidenced the presence of alkaloid compounds, for example, indicain and plantagonin [1]. The terpenoid compounds isolated from the leaves and leaf wax of *Plantago sp*. were loliolid, ursolic acid, oleanolic acid, and sitosterol acid 18 β-glycyrrhetinic. Another important class in *Plantaginaceae* is that of iridoid glycosides; aucubin was noticed as the main iridoid glycoside in *Plantago major*—this species may also contain small quantities of asperuloside, majoroside, 10-hydroxymajoroside, 10-acetoxymajoroside, catapol, gardoside, geniposidic acid, melittoside [1,8]. The iridoid glycosides content from *Plantago lanceolata* attain 2–3% (*w*/*w*); the main compounds found here are acucubin, catalpol, asperuloside, globularin, and desacetylasperuloside-acid methyl ester [11,14]. Generally, iridoids are oxygenated monoterpenoids, derived from geraniol [15]; these compounds play a major role in plant defense against herbivores, insects [16], ambient stress (temperature, drought) [17], and are respectively in control of interactions between plant roots and mycorrhiza [15]. Other important compounds in *Plantago* sp. are soluble polysaccharides, mucilage-type polymers based on sugars, and uronic acids, respectively [10,11,12]. The main sugars in polysaccharide fraction hydrolysate were arabinose, rhamnose, galactose, and galacturonic acid [12]. Particularly, the seeds of *Plantago* sp., namely Psyllium, contain xylose, arabinose, galacturonic acid, galactose, glucuronic acid, rhamnose, galactose, glucose, and a highly esterified pectin polysaccharide, acidic arabinogalactan, composed of arabinose, galactose, rhamnose, and galacturonic acid [1]. Furthermore, the crude mucilage also revealed the copresence of protein compounds, following essential amino acids (the evidenced amino acids were lysine, histidine, arginine, aspartic acid, threonine, serine, glutamic acid, proline, glycine, alanine, valine, methionine, isoleucine, leucine, tyrosine, and phenylalanine) [10]. The main fatty acids identified in *Plantago sp.* are lignoceric acid, palmitic acid, stearic acid, oleic acid, linoleic acid, linolenic acid, myristic acid, 9-hydroxy-cis-11-octadecenoic acid, arachidic acid, and behenic acid [1,2]. Literature data also indicates *Plantago* sp. as containing important amounts of minerals and microelements, for example, K, P, Cl, Na, Mg, Fe, Mn, Sr, Sc, Ti, V, Cr, Co, Ni, Ga, As, Br, Rb, Mo, Ag, Au, and Sb essential and trace elements [13,18,19,20].

Regarding the biological activities [18,19,20,21,22,23], the alcoholic extracts of *Plantaginaceae* have been proven to exhibit antimicrobial [18,19,20], antiviral [21], antioxidant [23,24], and antitumor activities [24,25,26,27]. Antimicrobial behavior was manifested against: bacteria (such as *Staphylococcus aureus*, *Bacillus subtilis*, *Escherichia coli*, and *Salmonella typhimuium*); fungi (such as *Candida albicans*, *Candida galabrata*, *Candida krusei* [28]); as well as micromycetes (such as *Alternaria alternata*, *Pyrenophora teres*, *Pyrenophora tritici-repentis* [29], *Fusarium solani* [30], and *Ustilago scitaminea* [31]). Antioxidant activity [3,7,23] was attributed to contained secondary metabolites, mainly polyphenolics content as well as antitumor potency, proved on numerous human cancer cell lines (e.g., human gastric cancer cells, human solid tumor cell lines, human non–small-cell lung cancer, human colorectal cancer, and human renal cell carcinoma) [24,25,26,27]. Research performed on *Plantain* from Isparta, Turkey, by Bahadori et al. [7] reveals the existence of compounds such as verbascoside, phenylethanoid glycosides, phenolic acids (chlorogenic acid, rosmarinic acid), as well flavonoid glycosides (i.e., hesperidin, hyperoside) in the alcoholic extract, analyzed by LC–ESI–MS/MS [7]. Sanna and collab [8], in the studies performed on alcoholic extracts obtained from *Plantain* developed from germplasm of Sardinia, Italy, found that in the case of plants cultivated on three types of soils, the content of polyphenolic compounds attains a maximum level in leaves in January and April. Researchers found in the alcoholic extract of *Plantain* leaves compounds such as chlorogenic acid, neo chlorogenic acid, cryptochlorogenic acid, and verbascoside. Luteolin was found only in an ethanolic extract from *Plantain* leaves harvested in July.

Luteolin derivates were proved able to reduce gastric acid synthesis, via smooth muscle inhibition, while caffeic acid normalized gall bladder function. The mucilage fraction from *Plantago lanceolata* has major therapeutic importance because it helps the regeneration of tissue damaged by forming a protective layer, under which the diseased tissue is regenerated [32,33,34].

Based on the scientific data presented in the literature, the present work aimed to study the biological activities of three extracts obtained from Plantain. In this regard, three fractions derived from *Plantain*, the polysaccharidic fraction (PP), the flavonoidic fraction (PF), and the iridoidic fraction (PI), respectively, were used. For these fractions, studies “in vitro” to determine their antioxidant, antifungal (using common micromycetes and dermatophytes), and antitumor properties were performed. The polysaccharides fraction was used in studies regarding the prebiotic properties.

## 2. Materials and Methods

### 2.1. Plant Bioproducts

Three types of solid products were obtained from dry leaves of *Plantago lanceolata* L. (source: Romanian indigenous plants, cultivated at Orastie, Romania, and marketed by Fares Company as dry plants, voucher specimen deposited in ICCF *Plant Material Storing Room*). These plants, ground and sifted (mesh diameter: 1 mm), were used to obtain solid bioproducts, according to the methodology presented below, established by the National Institute for Chemical-Pharmaceutical R&D (ICCF Bucharest), Romania [20,35].

(a) A quantity of 500 g of plantain powder firstly was extracted with 5000 mL of distilled water, by boiling under reflux. The aqueous extract was concentrated at 250 mL at 50 °C, using a rotary evaporator (Heidolph, Schwabach, Germany); then, the concentrate was treated with 1250 mL of 96% methanol solvent. The resulting precipitate was then dissolved into 250 mL distilled water and then treated with 1250 mL methanol (the operation was repeated twice). The final precipitate was dried in the oven (Gallenkamp, UK) at 45 °C and resulted in a fine gray powder, which was considered as the polysaccharidic fraction (PP).
(b) The solid vegetal material from the first extraction was re-extracted with the me-thanolic solution resulting from the polysaccharides precipitation (approx. 4500 mL total volume) for 1 h, at boiling temperature under reflux, in a continuous stirring system operated at 300 rpm. The resulting methanolic extract was concentrated at solid residue at 50 °C using a rotary evaporator after that, the residue was dissolved into 250 mL of distilled water. The aqueous solution was further extracted (three times) with 250 mL ethyl acetate, at boiling, under reflux, and the combined ethyl acetate fractions were concentrated in a rotary evaporator at 50 °C. The residue obtained was dissolved into ethanol and precipitated with ethyl ether. The final precipitate was considered the flavonoidic fraction (PF).
(c) The three aqueous fractions resulting from (a) and (b) were combined and then filtrated on the active charcoal. The resulting aqueous extract was concentrated in a rotary evaporator at 50 °C and the obtained solid residue was considered the iridoidic fraction (PI).

The three *Plantain* extracts PP, PF, and PI were qualitatively analyzed by high-performance thin-layer chromatography (HPTLC) and X-ray diffraction (XRD). The biological properties of these extracts were assessed in vitro by studies regarding antioxidant, antifungal, and antitumor activity. Additionally, for the extract enriched in polysaccharides (PP), studies regarding the probiotic activity were performed.

### 2.2. Regents Used in HPTLC Analysis

Chemical solvents (i.e., methanol, ethanol, ethyl acetate, formic acid, acetic acid) as well as the reference products (i.e., hyperoside (>97%), rutin (min. 95%), kaempferol (95%), cosmosiin (97%), vitexin (>96%), cynaroside (95%), chlorogenic acid (>95%), caffeic acid (99%), gallic acid (98%), and kaempferol (97%)) were purchased from Fluka and Sigma-Aldrich Co (Bucharest, Romania); reference compounds were prepared as 10^−3^M solution in 70% ethanol solution.

### 2.3. Qualitative Determination

High-performance thin-layer chromatography studies were performed using specific solvent systems (ethyl acetate-glacial acetic acid-formic acid-water, 100:12:12:26) [11] in order to assess the polyphenols distribution in the three extracts (PP, PF, and PI).

Crystallographic properties of the three solid extracts were assigned with X-ray diffraction analysis (XRD), using a Rigaku SmartLab 9 kW diffractometer (Rigaku Corp., Tokyo, Japan), operated at 45 kV and 200 mA, Cu_Kα_ radiation 1.54059 Å, in 2θ/θ scanning mode, between 2 and 90° (2θ). The obtained diffractograms were analyzed using the dedicated software PDXL and were processed for graphical representation using the graphing software Origin 2016 Pro (OriginLab Corporation, Northampton, MA, USA).

### 2.4. Antioxidant Activity Estimation

The antioxidant activity (AA) was assessed by the chemiluminescence (CL) method with luminol, using a GLOMAX 20/20 luminometer, single reagent injector, Model E 5321-PROMEGA, operated at wavelength λ = 430 nm. For this purpose, a quantity of 0.01 g from each solid extract was dissolved into 3 mL dimethyl sulfoxide (DMSO) in an ultrasonic bath, obtaining three stock solutions that contain 3.33 mg/mL for each extract. These solutions were stored in the refrigerator at 5 °C until use. From these solutions (samples for analysis), aliquots of 10–100 μL were used in the chemiluminescence studies. Reagents: solution LH2 (5-amino-2,3-dihydro-1,4-ftalazindione), with concentration c = 2.5 × 10^−5^M, in DMSO; buffer solution TRIS HCl with c = 50 mM with pH = 8.5; H_2_ O_2_ with c = 30 mM. Samples for analysis. Reagent blank (witness): 200 μL LH2 + 750 μL buffer solution + 50 μL H_2_O_2_. The testing of each sample was made by adding the reagents in the following order: 200 μL LH2 + 700 μL buffer solution + 50 μL sample + 50 μL H_2_O_2_. The volume of the added sample was subtracted from the volume of buffer solution so that the final volume of all reagents (with the analyzed sample) was 1000 μL.

Antioxidant activity (%), by means of the oxygen free radicals scavenging, of used extracts was calculated using Relation (1):

(1) $$A.A.=\frac{\left(\mathrm{Iw}-\mathrm{Is}\right)\times 100}{\mathrm{Iw}}$$

with Iw and Is as the CL intensity measured 5 s from the beginning of the CL reaction for the witness (Iw) and for each plant extract containing the sample (Is), respectively.

All measurements were made in triplicate; the average values of them in all representations were used, with standard deviations.

### 2.5. Antifungal Activities

#### 2.5.1. Strains Used in Antifungal Studies

(a) micromycetes: *Aspergillus niger* ATTC 1015, (*A. niger*), *Aspergillus terreus* ATTC 1012 (*A. terreus*), *Penicillium citrinum* ATTC 10105 (*P. citrinum*), *Penicillium digitatum* ATTC 9849 (*P. digitatum*), *Penicillium* sp. 1, and *Penicillium* sp. 2 (the last two species were isolated from infected wood).
(b) dermatophytes: *Microsporum canis* ATTC 10214 (*M. canis*), *Trichophyton mentagrophytes* ATTC 18748 (*T. mentagrophytes*), *Microsporum gypseum* ATTC 24102 (*M. gypseum*), and *Scopulariopsis brevicaulis* ATTC 1102 (*S. brevicaulis*).

#### 2.5.2. Methodology Used in Antifungal Studies

All the fungus strains were cultivated on Petri plates with PDA (potato dextrose agar) by spore inoculation; after 7 days, the spores of each strain were inoculated in a Petri plate (90 mm diameter) on PDA. After 15 min, in each Petri plate, three discs of cellulosic sterile paper of 5 mm diameter were placed at equal distance, impregnated with 20 μL from each extract prepared at the point 2.4. Separately, the solvent effect (DMSO) was evaluated by placing three discs of cellulosic papers impregnated with DMSO in Petri plates. The Petri plates used in quantification of the DMSO effect were previously inoculated with each studied fungal strain. All the plates were incubated for 48 h at 25 °C; after that, the inhibition diameters were measured. All results were presented as the average of three measurements, with corresponding standard deviation.

### 2.6. Antitumor Activities

#### 2.6.1. Cell Viability Assay by Flow Cytometry Analysis

The cell viability was assessed by the ability of Propidium Iodide (PI) to pass through the membrane of dead cells interacting with the DNA of the nuclei and emitting red fluorescence light. THP1 cells (2 × 10^3^ cell/well) were seeded in 96-microwell plates and treated with different concentrations of each of the PP, PF, and PI extracts at different concentrations (i.e., 0.05 mg/mL; 0.1 mg/mL; 0.2 mg/mL; 0.5 mg/mL; 1 mg/mL) for 24 h. Each solid extract was dissolved in a culture medium with 0.01% DMSO. The cells without PI staining were used as a negative control. Tumor cells THP-1 either untreated or treated with *Plantago* bioproducts for 24 h were incubated for 10 min with a 2 μg/mL solution of PI on ice in the dark. A total of 10,000 cells/sample were acquired using a BD Canto II flow cytometer. The flow cytometric analysis was performed using DIVA 6.2 software (Becton Dickinson Immunocytometry Systems, San Jose, CA, USA) to discriminate viable cells (FITC − PI^−^) from necrotic cells (FITC + PI).

#### 2.6.2. Cell Cultures

A leukemic THP-1 cell line (ATCC TIB 202) was used in experiments regarding the antitumor properties. This cell line was grown in RPMI 1640 medium, supplemented with the following reagents: 10% heat-inactivated fetal bovine serum (FBS); 2 mM glutamine; 100 U/mL penicillin, and 100 μg/mL streptomycin, at 37 °C in a humidified incubator with 5% CO_2_ atmosphere. Cultures were maintained by the addition of fresh medium or the replacement of medium every 2 to 3 days. Before treatment, the cell cultures were centrifugated and resuspended at 4 × 10^5^ viable cells/mL.

#### 2.6.3. Apoptosis Assay—Annexin V-FITC/PI Double Staining

The apoptosis assay was carried out with the Annexin V-FITC kit using the manufacturer’s protocol [36,37,38]. Cells were washed with cold PBS three times and resuspended in 1 mL PBS. To determine the rate of apoptosis, the treated and untreated 1 × 10^5^ cells/mL were resuspended in cold binding buffer and stained simultaneously with 5 μL FITC-Annexin V (green fluorescence) and 5 μL propidium iodide (PI) in the dark at room temperature for 15 min. Then, 400 μL of Annexin V binding buffer was added and 10,000 cells/per sample were acquired using a BD Canto II flow cytometer. The analysis was performed using the DIVA 6.2 software to discriminate viable cells (FITC − PI^−^) from necrotic cells (FITC − PI^+^) and early apoptosis (FITC + PI^−^) from late apoptosis (FITC + PI).

### 2.7. Prebiotic Activities

#### 2.7.1. Microorganisms

In prebiotic activities, we use four types of strains, respectively: *Lactobacillus plantarum* ATTC 8014 (*L. plantarum*), *Lactobacillus casei* ATTC 393 (*L. casei*), *Lactobacillus reuteri* ATTC 55730 (*L. reuteri*), and *Saccharomyces cerevisiae* (*S. cerevisiae*) (isolated from commercially Dr. Oetker bakery yeast). *Lactobacillus* species were incubated for 24 h on a liquid MRS medium without agar at 37 °C. *Saccharomyces cerevisiae* was grown on the specific media YPG.

#### 2.7.2. Methodology Used in Prebiotic Activities

For screening activities, 24-well microplates were used; for *Lactobacillus* sp. we used liquid MRS medium with or without dextrose; for *Saccharomyces* sp. we used YPG medium with or without dextrose. Into each well of the plate, the same volume of MRS or YPG medium with or without dextrose was distributed. In each well, a serial dilution was made with a volume of 0.25 mL sterile water (in the case of culture medium with dextrose) or with sterile stock solution, which contains PP or inulin. The solutions which contain polysaccharides, inulin, or dextrose were made in sterile water. Each row of the plate had the following concentrations of prebiotics (μg/mL): 5000; 1000; 200; 40; 8; and 1.6. In each well, the inoculum of the studied probiotic strain was evenly distributed, at the rate of inoculation of 1:10, from each probiotic strain, which contains 4 × 10^8^ CFU/mL. As a reference, the culture mediums MRS (for *Lactobacillus* sp.) or YPG (for *Saccharomyces* sp.) were used. The influence of PP, inulin, and dextrose on each probiotic microorganism was studied by replacing dextrose from MRS or YPG with PP or inulin, using serial dilutions. All tests were made in three replications, and each measurement was performed three times. The OD (optical density) of the well plate was measured after 24 h and 48 h at 620 nm using a DYNEX plate reader (DYNEX Technologies, MRS, VA, USA).

## 3. Results

### 3.1. Plant Bioproduct Characterization

**Figure 1:**
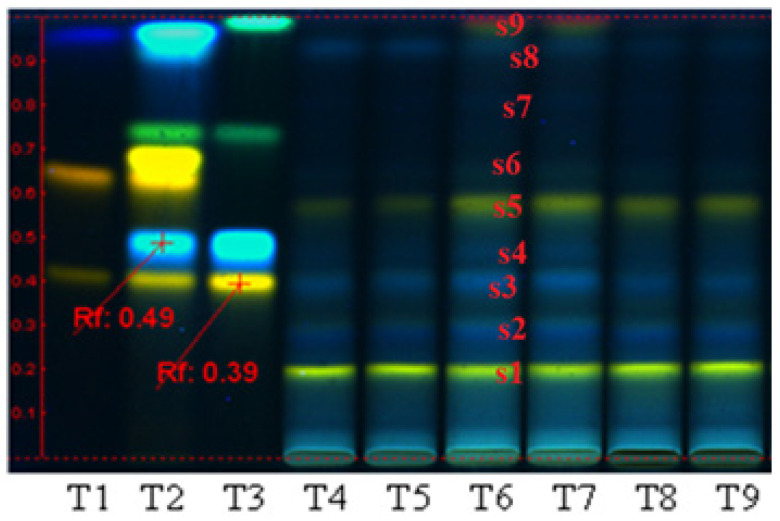
Qualitative assessment of polyphenolic compounds distribution in the three extracts obtained from *Plantago lanceolata*, by HPTLC analysis. T1: quercetin-3-O-rutinoside/rutin, quercetin-3-O-galactoside/hyperoside and gallic acid; T2: quercetin-3-O-rutinoside/rutin, chlorogenic acid, quercetin-3-O-galactoside/hyperoside, luteolin-7-O-glucoside/cynaroside, apigenin-8-C-glucoside/vitexin, caffeic acid; T3: rutin, chlorogenic acid, apigenin-7-O-glucoside/cosmosiin and kaempferol; T4–T5: polysaccharides fraction PP; T6–T7: polyphenols fraction PF; T8–T9: iridoids fraction PI.

Quantification of polyphenolic compounds in the three extracts (PP, PF, and PI) by HPTLC (Figure 1) reveals that the polyphenols were distributed in all the three fractions. This analysis indicates some differences regarding the quality distributions of polyphenolic compounds between the three bioproducts; therefore, in PP and PI, the bands obtained appear to be almost identical, and most probably the PP extract contains iridoidic compounds, too. Regarding the PF extract, this fraction contains luteolin and hyperoside derivatives compounds (s1, s2), rutin (s3), chlorogenic acid (s4) hyperoside (s5), cynaroside (s6), caffeic acid (s8), and kaempferol (s9).

The PI fraction may contain luteolin and hyperoside derivatives (s1, s2), rutin (s3), traces of hyperoside (s5), and chlorogenic acid (s4). The quantitative analyses performed on the same plant (i.e., plant source: Fares, Orastie, Romania), and already reported [4,6,11,35], showed that the content of polyphenolic compounds in the bioproducts separated from aqueous media (polysaccharidic fraction) can reach 0.89% GAE/g dry plant [4]. Regarding the bioproducts separated from alcoholic media, from the same source of raw materials, the reported data showed that the crude extract may contain 0.89% total polyphenols [6]. The polyphenolcarboxilic acids represent 0.7% and flavonoids 0.63% [6]. The analysis performed on the bioproducts obtained from the alcoholic fractions by electrospray ionization mass spectrometry indicated the presence of apigenin, luteolin, aucubin, chlorogenic acid, catalpol, and luteolin 7-O-β glucosides as polyphenolic compounds. Regarding iridoidic compounds, Plantain may contain iridoid glycosides such as aucubin and catalpol in quantities of a maximum 3%/plant [11].

**Figure 2:**
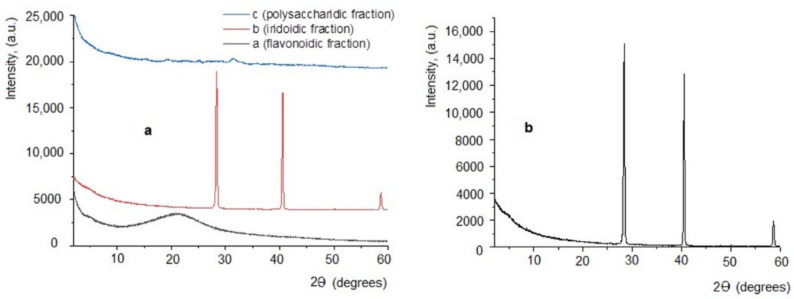
X-ray diffraction analysis. (**a**) Diffractograms obtained for extracts of *Plantago lanceolata*: a = PP; b = PI; c = PF; (**b**) Diffractogram details from the sample PI (iridoidic fraction).

Results obtained from X-ray diffraction analysis (Figure 2a,b) revealed the fact that the three extracts obtained are different: the PF fraction has a classical diffractogram, specific to amorphous substances (without crystalline structure) (Figure 2a, black line). Regarding the PI extract, the obtained diffractogram indicates a crystalline structure, with two high-intensity diffraction peaks, located at 14.178 and 20.2693 theta and at 29.2959 theta, respectively (Figure 2a red line and details in Figure 2b), and interplanar distances ranging between 1.5742 to 18.0627 Å (Table 1). As for the PP fraction, it has a weakly crystalline structure, indicating a low-intensity diffraction peak located at 15.625 theta (Figure 2a, blue line), and other very low diffraction peaks situated at 7.5, 9.75, and 12.5 theta.

### 3.2. Antioxidant Properties

**Figure 3:**
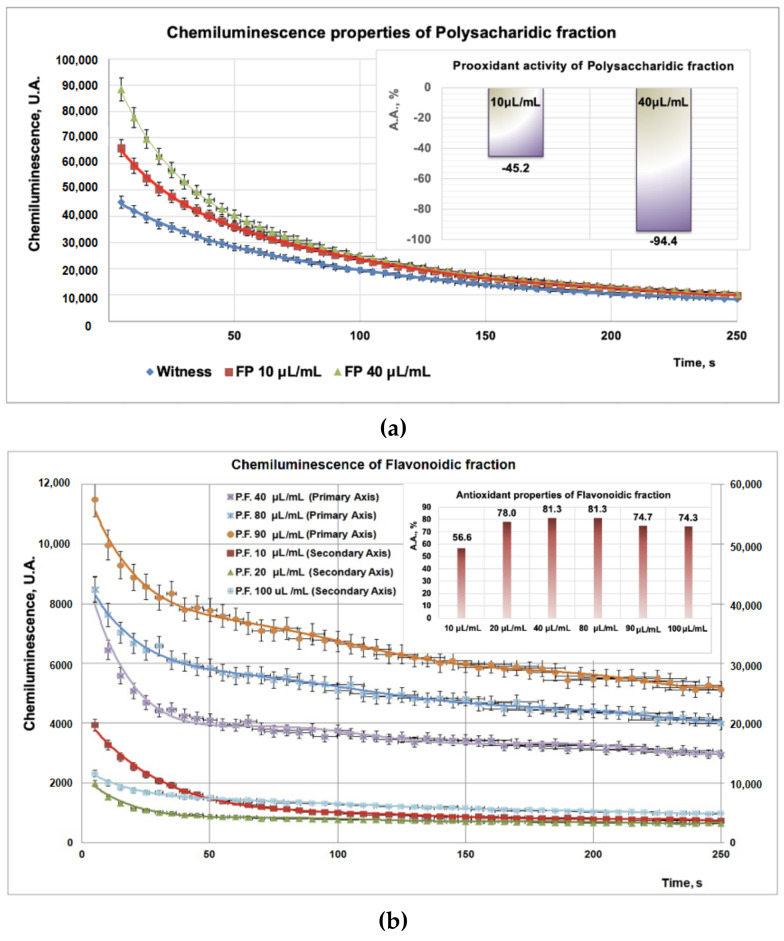

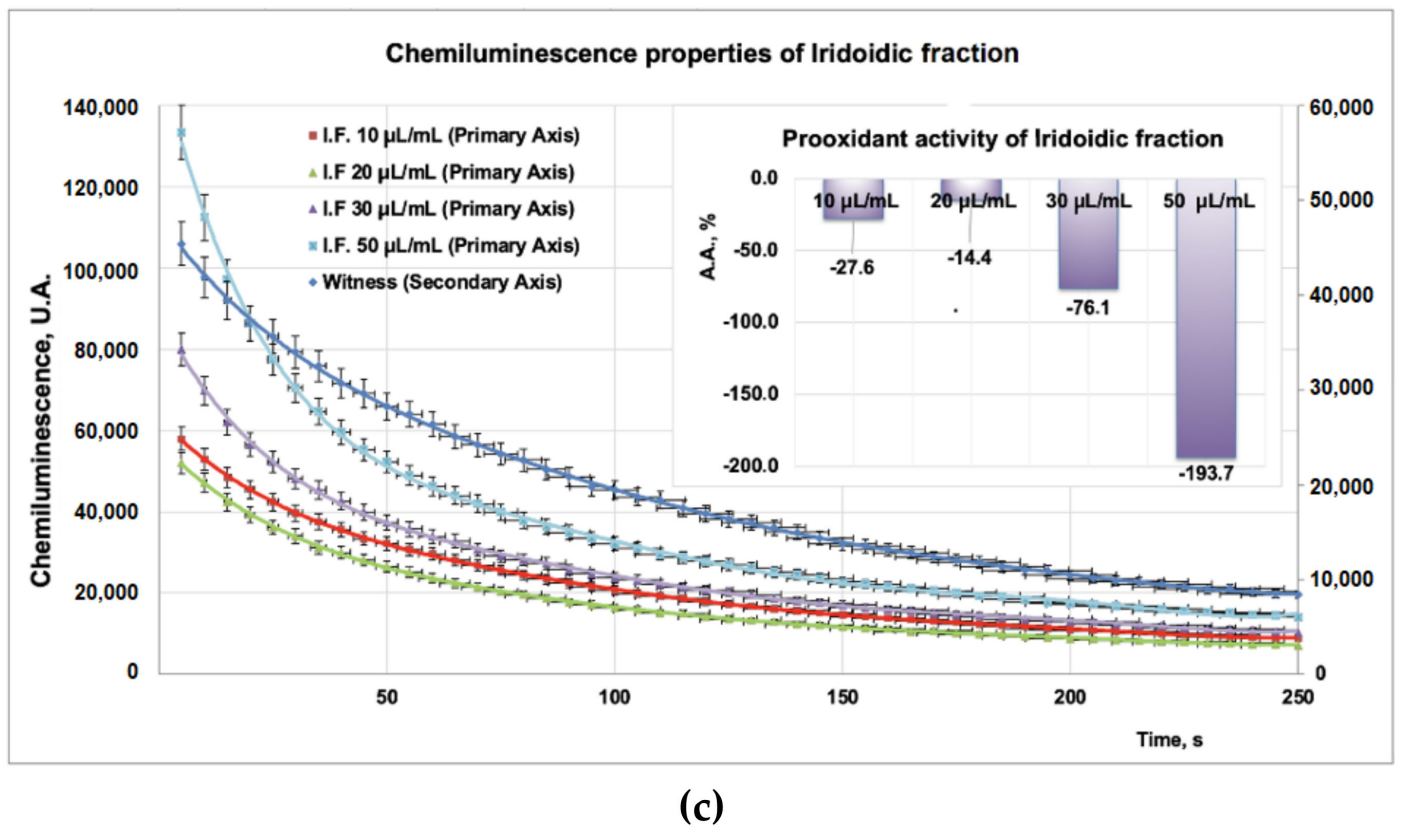
(**a**). Studies regarding chemiluminescence/prooxidant properties of PP fraction. (**b**) Studies regarding chemiluminescence/antioxidant properties of PF fraction (with the same witness as in (**a**)). (**c**) Studies regarding chemiluminescence/prooxidant properties of PI fraction.

Due to the high sensitivity and rapidity, the addition of compounds into a chemiluminescence (CL) system leads to the reduction of the CL emission, thus scavenging the reactive oxygen species. Experimental data on PP/PF/PI fractions obtained from CL studies revealed a strong antioxidant effect for the PF fraction (Figure 3b inset) for all tested doses (10–100 μL), whereas, for the PP (tested doses, 10–40 μL) and PI (tested doses, 10–50 μL) samples, a strong prooxidant effect was observed (Figure 3a inset, Figure 3c inset). Moreover, the prooxidant effect of the PP fraction (Figure 3a inset) is more pronounced than that of the PI fraction (Figure 3c inset).

### 3.3. Antifungal Properties of Plantago Bioproducts

**Figure 4:**
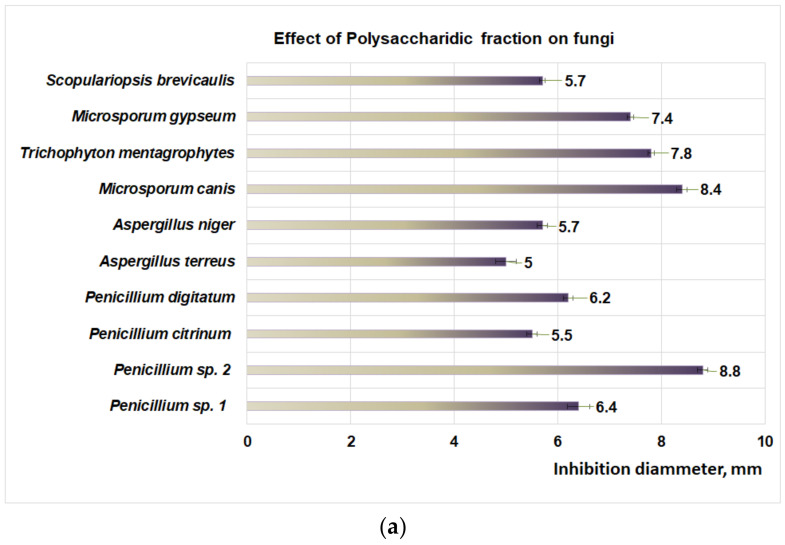

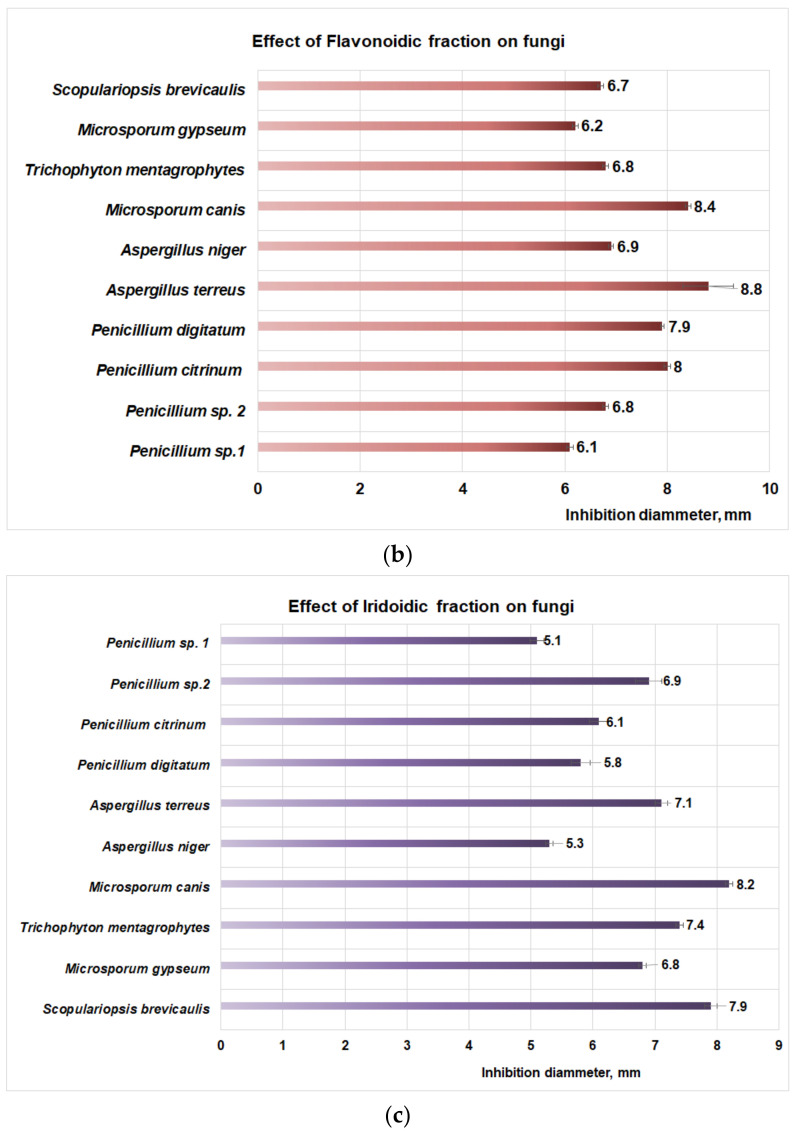
(**a**) The effect of polysaccharidic fraction on different fungal strains. (**b**) Effect of flavonoidic fraction on different fungal strains. (**c**) Effect of iridoidic fraction on different fungal strains.

Studies performed with DMSO reveal that after 48 h the aprotic solvent did not have an inhibitory effect on fungal strains involved in this study. Regarding the action of the PP bioproduct on fungal dermatophytes (Figure 4a), the moderate action is obtained at 48 h of exposure for *M. canis* (8.4 mm inhibition diameter), followed by *T. mentagrophytes* (7.8 mm inhibition diameter), *M. gypseum* (7.4 mm inhibition diameter), and *S. brevicaulis* (inhibition diameter 5.7 mm). In the case of micromycetes, the PP fraction has a moderate effect on *Penicillium* sp. *2* (inhibition diameter of 8.8 mm) and *Penicillium* sp. *1* (inhibition diameter of 6.4 mm) (Figure 4a). The flavonoidic fraction acts similarly on dermatophytes (Figure 4b), hence moderate activity is obtained for *M. canis* (inhibition diameter 8.4 mm), followed by *T. mentagrophytes* (6.8 mm inhibition diameter), *S. brevicaulis* (6.7 mm diameter of inhibition), and *M. gypseum* (6.2 mm inhibition diameter).

Moderate effects of the PF fraction were also observed for micromycetes (Figure 4b) such as *A. terreus* (inhibition diameter 8.8 mm) followed by *P. citrinum* (inhibition diameter 8 mm), *P. digitatum* (inhibition diameter 7.9 mm), *A. niger* (inhibition diameter 6.9 mm), *Penicillium* sp. 2 (inhibition diameter 6.8 mm), and *Penicillium* sp. 1 (inhibition diameter 6.1 mm).

The studies regarding the effect of PI fraction on fungal dermatophytes (Figure 4c) revealed a moderate action on *M. canis* (inhibition diameter 8.2 mm), followed by *S. brevicaulis* (inhibition diameter of 7.9 mm), *T. mentagrophytes* (7.4 mm inhibition diameter), and *M. gypseum* (diameter of inhibition of 6.8 mm). In the case of micromycetes, the PI fraction (Figure 4c) showed a moderate effect on *A. terreus* (inhibition diameter 7.1 mm), followed by *Penicillium* sp. 2 (inhibition diameter of 6.9 mm), *P. citrinum*, (diameter of inhibition of 6.1 mm), *P. digitatum* (inhibition diameter of 5.8 mm), *A. niger* (inhibition diameter of 5.3 mm) and *Penicillium* sp. 1 (inhibition diameter of 5.1 mm).

### 3.4. Antitumor Properties of Plantago Bioproducts

**Figure 5:**
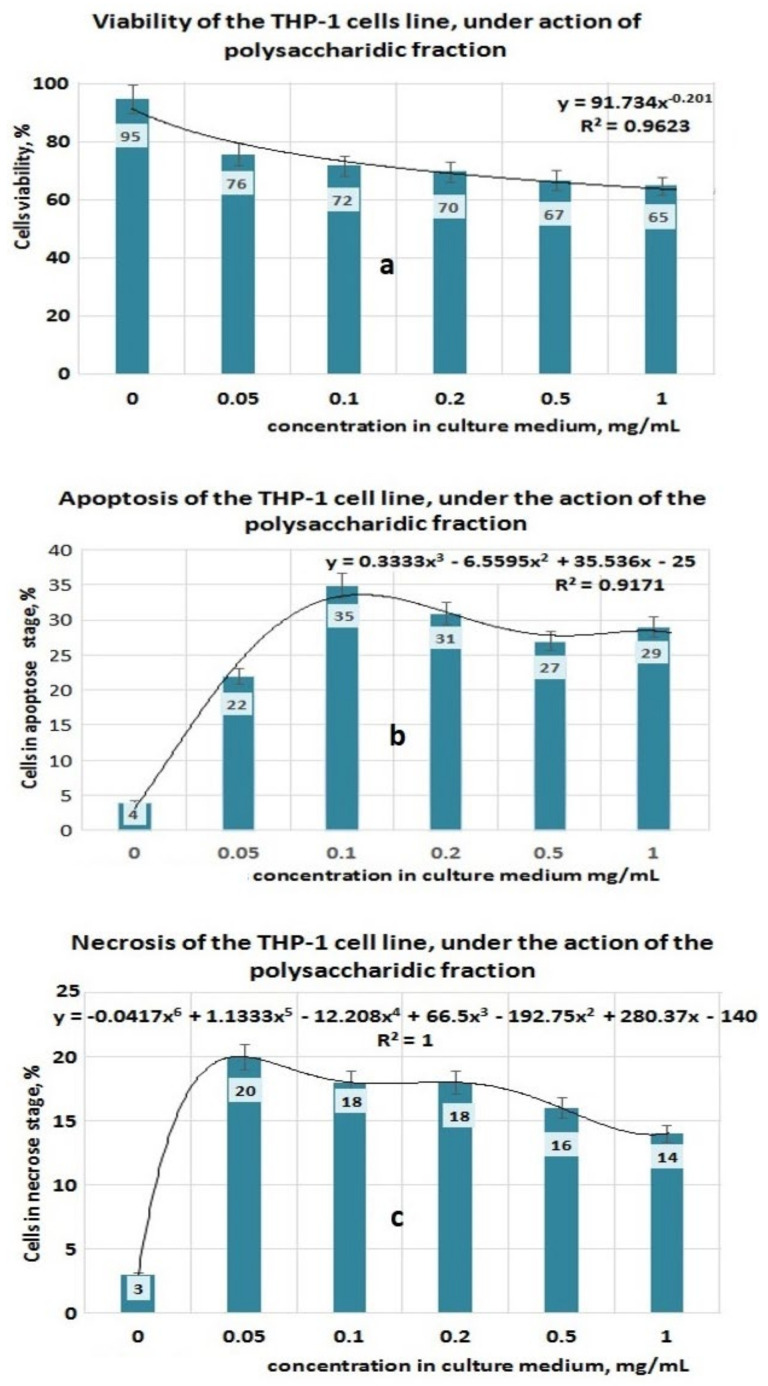
Effect of exposure at PP fraction on THP–1 cell lines. (**a**) Viability of THP–1 cell line under action of PP fraction; (**b**) Apoptosis of THP–1 cell line under action of PP fraction; (**c**) Necrosis of THP–1 cell line under action of PP fraction.

The study performed on the THP-1 cell line regarding the action of PP (Figure 5a–c) showed that under the action of this bioproduct, the viability of the cells decreased with increasing PP concentration in the culture medium (Figure 5a), followed by a decreasing power function so that at 1 mg PP/mL the viability of the cells became 65%. The apoptosis values obtained for the same PP concentrations in culture media indicated that up to 0.1 mgPP/mL, the apoptotic process increased up to 35% and after this concentration the level of apoptotic cells decreased, even if the PP concentration in culture media increased. The apoptotic process of THP-1 cells that have been exposed to the action of PP bioproduct can be approximated with a polynomial function of degree three, with a good correlation coefficient (0.9171) (Figure 5b). The necrosis process appears to attain maximal value (20%) at 0.05 mg PP/mL (Figure 5c), and this fact suggests that most of the cells that are in the apoptotic state pass into the necrosis stage. By matching all experimental data obtained for the PP bioproduct, it can be estimated that the mechanism of destroying the THP-1 cell line is complex and the apoptosis process affects it to a large extent. The antitumor effect of PP bioproduct can be due to some polyphenolic compounds that pass in the PP fraction when it is separated from the *Plantain* leaves.

**Figure 6:**
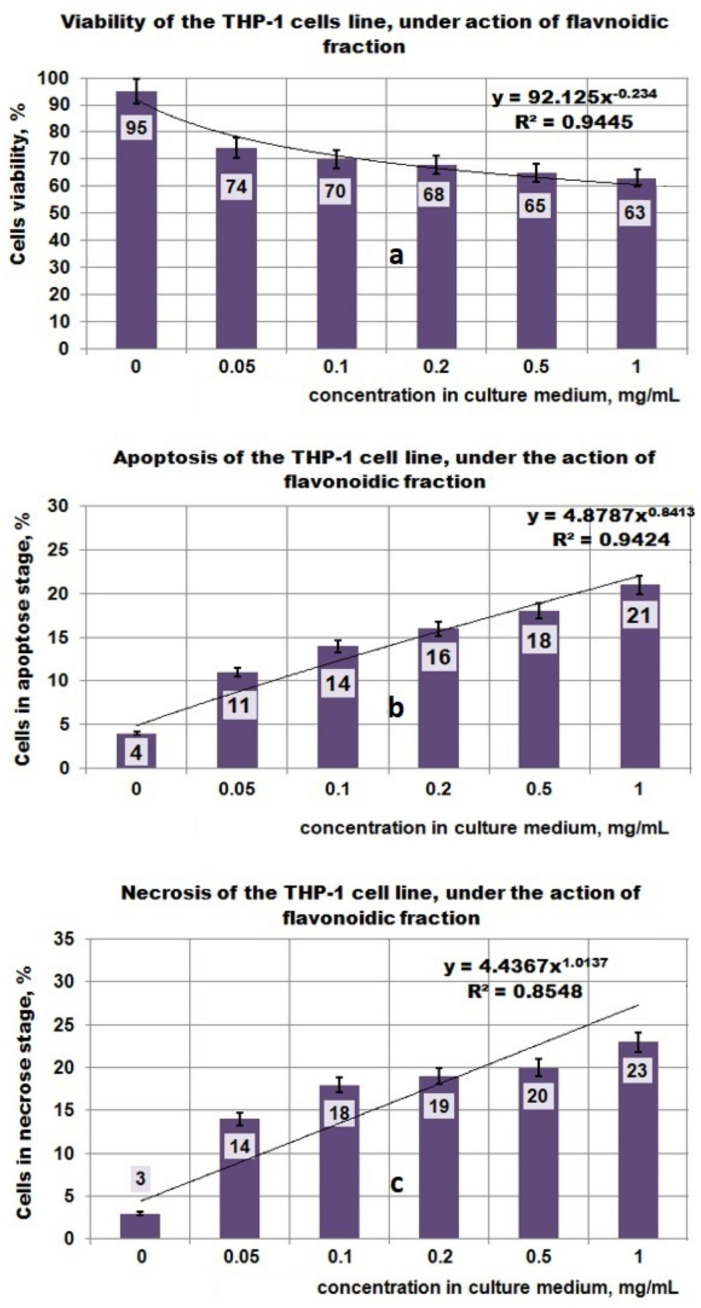
Effect of exposure at PF fraction on THP–1 cell lines. (**a**) Viability of THP–1 cell line under action of PF fraction; (**b**) Apoptosis of THP–1 cell line under action of PF fraction; (**c**) Necrosis of THP–1 cell line under action of PF fraction.

Investigations performed with the PF fraction (Figure 6a–c) indicated that for the increasing concentration of PF in the culture medium, the cell viability decreased, and reached 63% at 1 mg PF/mL. This process can be mathematically characterized by a decreasing power function (Figure 6a), as in the case of the PP fraction as well. At the same time, by increasing the PF concentration in the culture medium, the cells found in the apoptosis and necrosis stages increased (Figure 6b,c) and both processes can be mathematically characterized by an increasing power function (Figure 6a). Based on a mathematical model established in this study, it can be observed that the necrosis process is relatively faster in comparison with apoptosis. Thus, at 1 mg PF/mL, 21% of THP-1 cells were in the apoptosis stage and 23% in the necrosis stage. The mechanisms appear to be complex, involving the apoptotic and necrosis processes. Interesting for this type of bioproduct is the fact that all investigated parameters (i.e., viability, apoptosis, necrosis) can be mathematically characterized by the same type of function (i.e., a decreasing or increasing power function).

**Figure 7:**
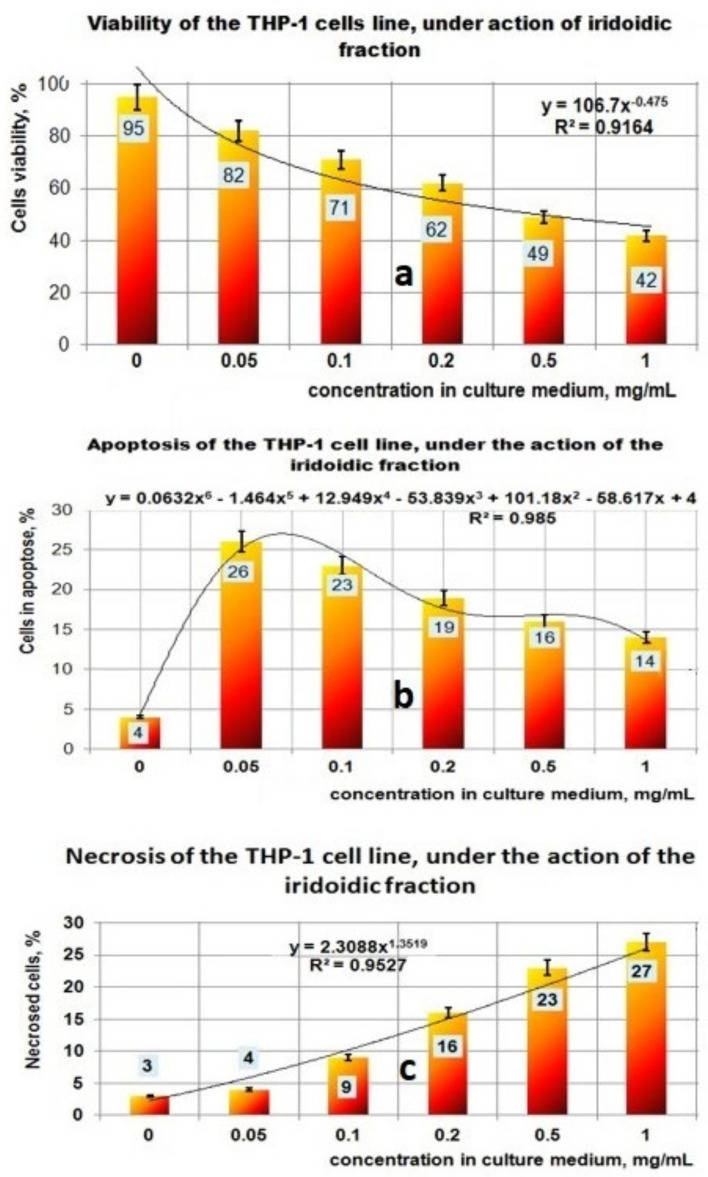
Effect of exposure at PI fraction on THP–1 cell lines. (**a**) Viability of THP–1 cell line under action of PI fraction; (**b**) Apoptosis of THP–1 cell line under action of PI fraction; (**c**) Necrosis of THP–1 cell line under action of PI fraction.

The results obtained in the case of the fraction which contains iridoidic compounds (Figure 7a–c) are much more spectacular. Under the action of PI bioproduct in the range of (0–1) mg/mL, the viability of the THP-1 cell line decreased from 95% to 42%; this process can be mathematically approximated with a decreasing power function (Figure 7a). Regarding the influence of the PI fraction on apoptosis, the results indicated a maximum effect at 0.05 mg/mL when 26% of the THP-1 cell line found it in the apoptotic stage (Figure 7b). At a concentration greater than 0.05 mg PI/mL, the apoptotic process decreased until 14% for 1 mg PI/mL. This apoptotic process due to the presence of PI bioproduct in the studied concentration range can be well described by a polynomial function of degree six (Figure 7b). The necrosis process of THP-1 cells due to the presence of PI in the culture medium is characterized by an increasing power function, and ranged from 3% for 0 mg PI/mL to 27% at 1 mg PI/mL (Figure 7c).

### 3.5. Prebiotic Activity of Plantain Bioproducts

**Figure 8:**
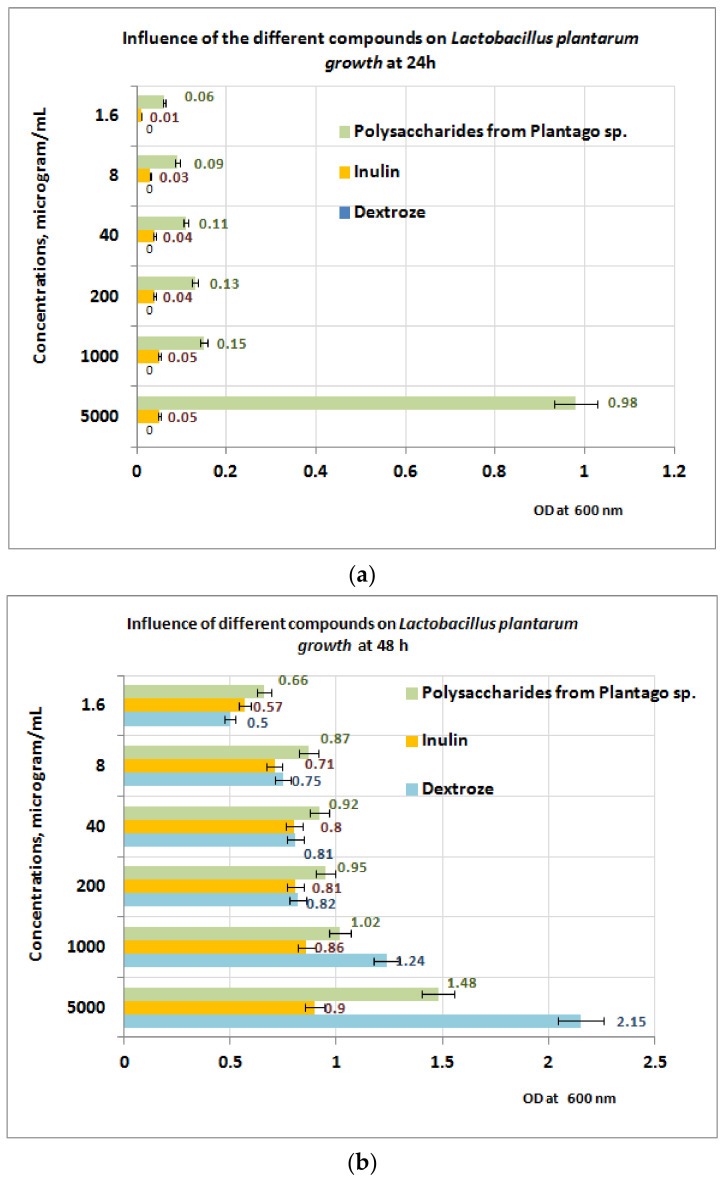
(**a**) Influence of PP on *Lactobacillus plantarum* growth at 24 h. (**b**) Influence of PP on *L. plantarum* growth at 48 h.

Experiments regarding prebiotic activity were performed on the PP extract only, because both PF and PI extracts contain large quantities of polyphenolic compounds without prebiotic effects. The growth of *L. plantarum* after 24 h of exposure appears to be well influenced by the presence of PP fraction, in comparison with inulin (small growth) and dextrose (no growth) (Figure 8a). At 24 h, the best results are obtained at a concentration of 5 mgPP/mL (OD = 0.98) for *L. plantarum*.

At 48 h of exposure, the measurements performed reveal that in the concentration range of 1–5 mg/mL the *L. plantarum* growth is best influenced in the presence of dextrose, followed by PP bioproduct (Figure 8b). For a concentration range situated between (1.6 ÷ 200) μg/mL, the best results regarding the *L. plantarum* growth were obtained for PP bioproduct followed by dextrose and inulin. The obtained results are probably due to the intensification of the depolymerization processes of the PP bioproduct, under the action of metabolic compounds resulting from *L. plantarum* growth, processes followed by the appearance of monosaccharides in culture medium, which are more accessible for probiotic strain.

**Figure 9:**
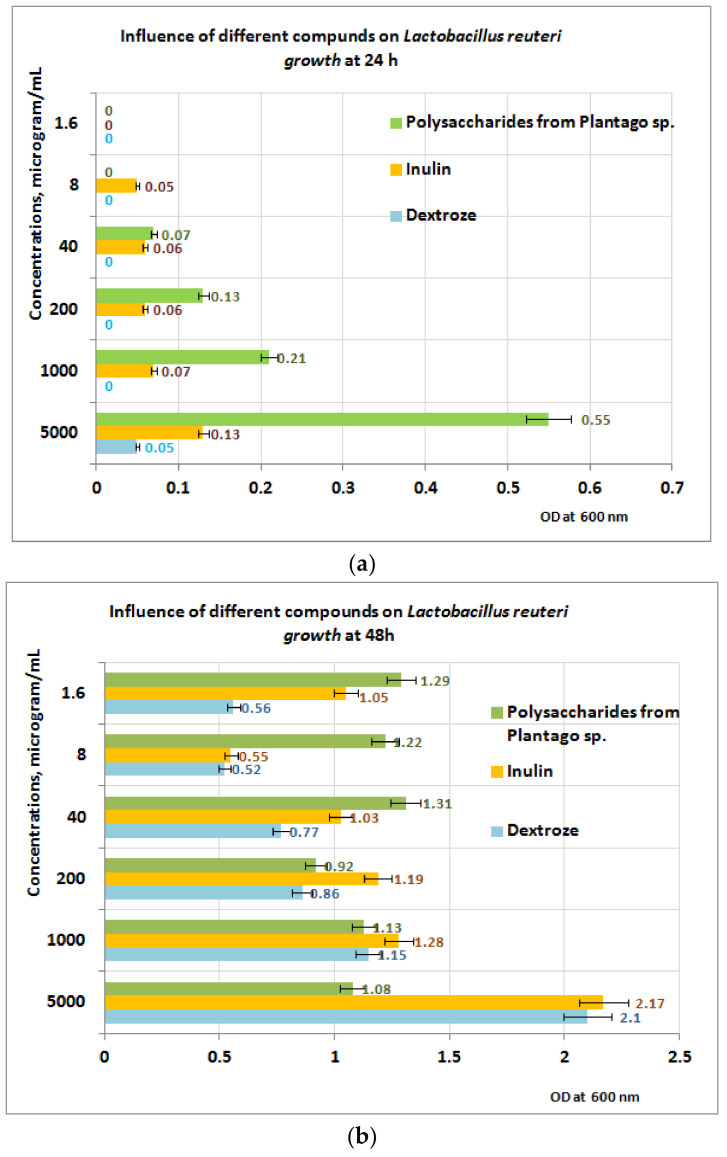
(**a**) Influence of PP on *L. reuteri* growth at 24 h. (**b**) Influence of PP on *L. reuteri* growth at 48 h.

In the case of *L. reuteri* (Figure 9a), the best results appear to be obtained after 24 h in favor of the PP fraction, in the PP concentration range of (40–5000) µg/mL. After 48 h of exposure to PP (Figure 9b), the measurements performed indicate that in the concentration range of (1 ÷ 5) mg PP/mL, the growth is best influenced by the presence of inulin, followed by dextrose and PP (Figure 9b). At 200 μg PP/mL, the best results are obtained for inulin, followed by PP and dextrose. If the concentration range of the PP fraction is between (1.6 ÷ 40) µg/mL, the best results regarding the *L. reuteri* growth are obtained in the presence of PP followed by inulin and dextrose. These results are probably due to the absence of metabolic compounds of the *L. reuteri*, without influence or with small influence regarding the depolymerization processes of the PP bioproduct.

**Figure 10:**
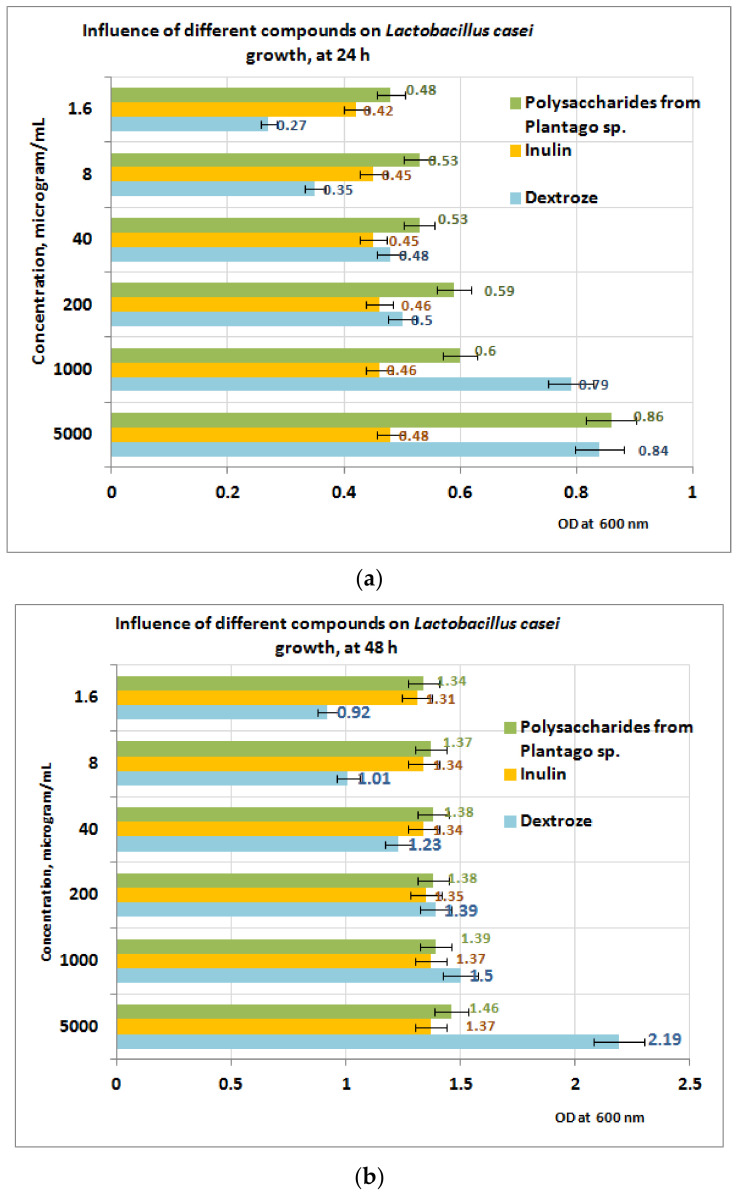
(**a**) Influence of PP on *L. casei* growth at 24 h. (**b**) Influence PP on *L. casei* growth at 48 h.

In the case of *L. casei* (Figure 10a,b), the growth after 24 h of exposure to PP appears to be well influenced by the presence of the PP fraction compared to inulin and dextrose (Figure 10a). At 24 h, the best results are obtained at a concentration of 5 mg PP/mL (OD = 0.86). After 48 h of exposure, the measurements performed indicate that in the concentration range of (1 ÷ 5) mg PP/mL the growth of the *L. casei* is best influenced in the presence of dextrose, followed by PP and inulin (Figure 10b). In the concentration range situated between (1.6 ÷ 40) μg/mL, the best results are obtained for PP bioproduct, followed by inulin and dextrose.

**Figure 11:**
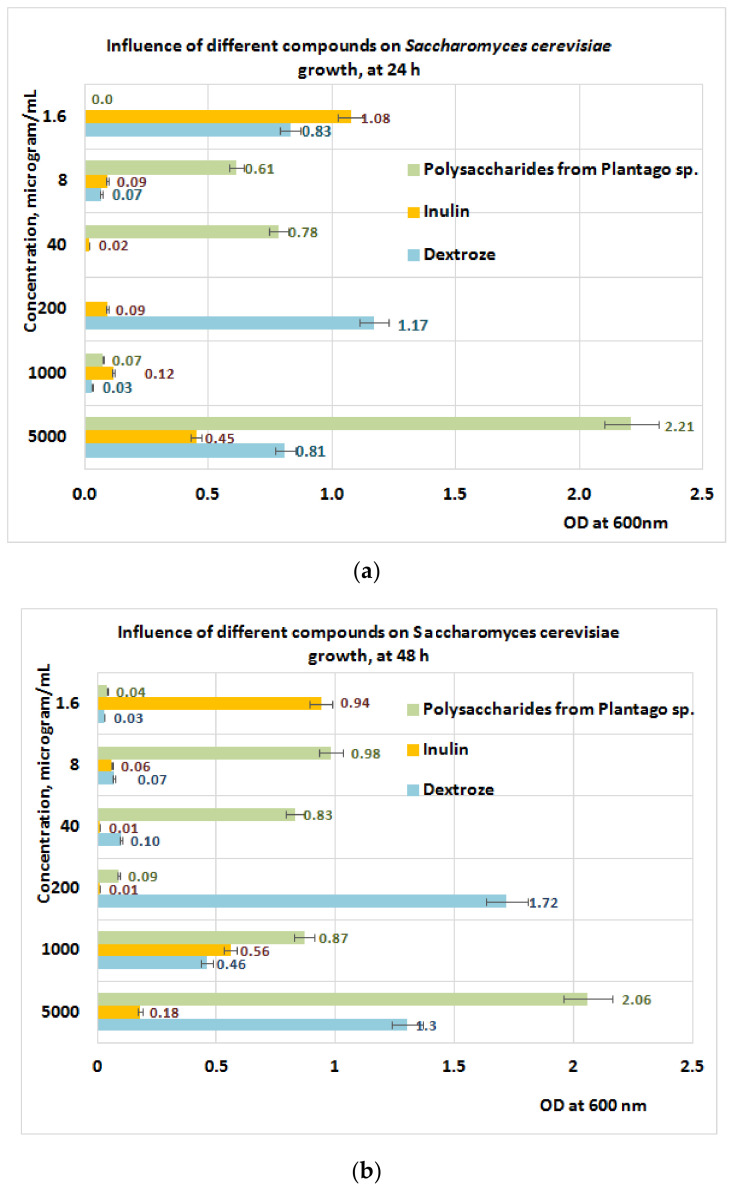
(**a**) Influence of PP on *S. cerevisiae* growth at 24 h. (**b**) Influence of PP on *S. cerevisiae* growth at 48 h.

Studies performed on *S. cerevisiae* (Figure 11a,b) after 24 h of exposure to PP revealed a positive influence of PP fraction regarding its growth in the concentration range of (8 ÷ 40) μg/mL, when the best results are obtained for the PP fraction followed by inulin and dextrose (Figure 11a). The data obtained after 48 h of exposure indicate a slow growth of *S. cerevisiae* at the same concentration range of PP (Figure 11b).

## 4. Discussion

Analyses performed with the HPTLC technique show that the three extracts, codified PP (polysaccharidic fraction), PF (flavonoidic fraction), and PI (iridoidic fraction), contain variable amounts of the main polyphenolic compounds in *Plantaginaceae*, caffeic acid derivates and luteolin derivates. X-ray diffraction analysis on the three bioproducts also indicated an amorphous structure for the PF fraction, but a crystalline structure for the PI and PP fractions (specifically with two high-intensity diffraction peaks, located at 14.178, 20.2693, and 29.2959 theta in the case of the PI fraction, and a lower intensity diffraction peak located at 15.625, 7.5, 9.75, and 12.5 theta in the case of the PP fraction).

The antioxidant activity of the PF fraction is due to the presence of compounds such as luteolin and hyperoside derivatives compounds, rutin, chlorogenic acid, hyperoside, cynaroside, caffeic acid, and kaempferol [39] all evidenced by HPTLC analysis. These results are in agreement with results obtained by Abate and collab [40] who reported that the methanolic extract of *Plantain* leaves may contain 4-dihydroxyphenylacetic acid, (+)-catechin, pyrocatechol, vanillin, verbascoside, epicatechin, taxifolin, hesperidin, rosmarinic acid, pinoresinol, eriodictyol, and kaempferol. The same authors report that in the alcoholic extract there exists significant levels of kaempferol (43.64 mg/g), luteolin (5.35 mg/g), apigenin (8.27 mg/g), p-hydroxybenzoic acid (149.46 mg/g), 2,5-dihydroxybenzoic acid (16.20 mg/g), proto-catechuic acid (103.48 mg/g), vanillic acid (411.52 mg/g), gallic acid (212.01 mg/g), apigenin (184.38 mg/g), (cymaroside) luteolin-7-O-glucoside (119.15 mg/g), and quercetin-3-O-glucoside (34.67 mg/g) [40] Regarding the flavonoidic compounds, in the same study the existence of compounds such as luteolin-7-O-glucuronide, luteolin, apigenin, luteolin-7-O-glucoside, and quercetin-3-O-D-galactopyranoside, 3, 5, 7, 4-tetrahydroxyflavonol, apigenin-6,8-di-C-glucoside, luteolin-7-O-glucoside, and 7-O-glucuronide-3′-glucoside, as well as quercetin-3-rutinoside, 7-O-glucuronide, and apigenin-7-O-glucoside was reported. Other studies [41] reported the presence of compounds such as 3,4-dihydroxyphenylacetic acid, catechin, pyrocatechol, vanillin, verbascoside, epicatechin, and taxifolin, hesperidin, rosmarinic acid, pinoresinol, eriodictyol, and kaempferol. Regarding the flavonoidic compounds, the same scientists found the existence of compounds such as luteolin-7-O-glucuronide, luteolin, apigenin, luteolin-7-O-glucoside, and quercetin-3-O-D-galactopyranoside. The pro-oxidant activity is due likely to the low level of polyphenolic compounds in the PP and PI fractions, and for this reason, these fractions are not able to act as ion scavengers.

However, these two fractions are not the same as we can see from the XRD analysis. The co-presence of polyphenolic compounds in the three types of bioproducts from *P. lanceolata* can also explain the antimicrobial and antitumor activity. Regarding the probiotic activity, this can be due to the good hydrolyzation of *Plantago* polysaccharides which are present in the units of galacturonic acid (Gal A), glucose (Glc), arabinose (Ara), and rhamnose (Rh). This fact can be responsible for the good metabolization of probiotic bacteria, in comparison with inulin. Regarding the elemental unit contents of the PP fraction, Lukova and collab [12] in studies performed on polysaccharidic fractions obtained from *Plantago lanceolata*, found that the fractions soluble in water contained as major units GalA (70.58%), Ara (29.42%), and traces of Rha. Another study performed by Kardosova in 1992 [40] indicates that the mucilages obtained from dry leaves of *Plantago lanceolata* contain D-Glc (21.9%), D-Gal (35.8%), L-Ara (26%), uronic acid (6.9%), D mannose (D-Man) (4.6%), and L-Rha (4.6%). Zhang and collab [42] found that the polysaccharides obtained by extraction with hot water at 80 °C from *Plantago* sp. contain units of Gal A (64.88%) and Ara (29.42%); these types of polysaccharides can stimulate the activity and growth of the gut microbiome. Other studies were performed by Lukova and collab. in 2020 [43]. on probiotic microorganisms such as *L. acidophilus*, *L. sakei*, and *L. brevis*, and revealed the probiotic activities of the polysaccharidic fraction obtained from *Plantago major*. This fraction contains GalA (55.38%), Glc (21.5%), Ara (9.88%), Gal (8.02), Rha (3.17%), and xylose (Xy) (2.05%). Bioproducts with polysaccharides obtained from *Plantago* species also contain macro- and microelements and did not show antimicrobial activities against *E. coli* and *S. aureus* [18]. Regarding the biological activities of this bioproduct, moderate activity against microfungi and the leukemic cell line can be due to iridoid glycosides [44]. The content of the bioproduct enriched in flavonoids found in this study (PF) is in agreement with other studies [5,7,45]. Regarding the antioxidant activity, similar results were reported by Lukova and collab. on alcoholic extract obtained from *P. lanceolata;* this extract contains polyphenolic compounds (17.37 mg GAE/g) that have antioxidant properties, evidenced by tests performed with DPPH (AA = 59%) [39].

Antimicrobial activities of different bioproducts obtained in various solvents are reported for microorganisms such as *E. coli*, *S. aureus*, *P. aeruginosa*, *K. pneumoniae*, *P. mirabilis*, *C. albicans*, and various *Streptoccosus* species as *S. aglactiae*, *S. pneumoniae*, *S. bovis*, *S. mutans*, *S. sobrinus*, *S. parasanguinis*, and *S. viridans* [46]. Ziarno and collab [47] in studies performed on aqueous extract obtained from *Plantago lanceolata,* which contains 41.84 mg GAE/g, did not find an inhibitory effect of the polyphenolic content on the bacterial population of *S. thermophilus* or *L. delbruecki* spp. bulgaricus in the field of concentrations ranging between 0.2–1.4% during 4 h of fermentation. If the concentration of aqueous extract increased to 3% in the culture media, the bacterial population decreased slightly with (0.5–0.6) logarithmic units for each microorganism involved in the study [47]. Beara and collab. in studies performed in vitro, put into evidence the antitumor properties of the alcoholic extract obtained from *P. lancelolata*; according to these scientists, cytotoxicity was observed on MRC-5 (IC560 = 551.69 μg/mL), HeLa (172.32 μg/mL), MCF-7 (142.78 μg/mL), and HT-9 (IC50 = 405.5 μg/mL) [5]. Chiang and collab. performed studies on leukemic cell line types HL-60, K562, CCRF-CEM, and P3HRI and revealed that the aucubin, ferulic acid, p-coumaric acid, and vanillic acid show low antileukemic activities (IC50 = (26–56 μg/mL)), whereas luteolin has a strong antileukemic activity (IC50 < 18 μg/mL) [48]. Weber et al. found that the caffeic acid derivates named plantamajoside, which are found in leaves of *Plantago lanceolata*, exhibit antitumor activities against HL-60 and P338 cell lines [49]. Experimental studies performed “in vitro” by Yang and collab. with these compounds (obtained by preparative chromatography) on HL-60 and P338 cell lines indicated a value of IC 50 for HL-60 and P338 greater than 100 μM [50]. Regarding the mechanism of action, other studies indicate that under the action of flavonoids (i.e., kaempferol) the activation of p53 is stimulated, and the level of proapoptotic proteins such as Bax and Bcl-2 is upregulated, favoring the beginning of apoptosis processes in leukemic cells [51,52]. The biological activity of PI products is due probably to iridoid glycosides such as aucubin, catalpol, or its derivatives, for which antimicrobial activities were reported against bacteria and fungi [20,40,46]. Other studies performed on catalpol and aucubin reported antimicrobial activities of these two compounds against *E. coli*, *E. faecalis* (MIC 512 μg/mL), *P. aeruginosa*, *S. aureus* (MIC = 256 μg/mL), *C. albicans* (MIC = 128 μg/mL and 256 μg/mL, respectively), *C. krusei* and *C. parapsilopsis* (MIC = 256μg/mL) [40,46]. These compounds exhibit cytotoxicity in K562 cell lines by inhibiting proliferation due to phase G1 from the cell cycle. Iridoid glycosides inhibit tumor proliferation by upregulation of p53 or p21 genes, which stops the cell cycle, as well by cell accumulation in the phase G0/G1 [44].

The analysis of the results obtained by the in vitro experiments performed on the THP-1 tumor cell line in this study led to the following aspects:

- the PP fraction reduced the cell viability of the THP-1 tumor cell line in a concentration-dependent manner. This PP fraction has antitumor properties which are sustained by its ability to induce apoptosis or necrosis of THP-1 tumor cells;
- in the case of the PF fraction, the antitumor effect seems to be higher, this being supported by the mathematical analysis of the results which shows a significant increase in both the apoptotic process and the necrosis that correlates with the decrease in viability;
- the fraction containing iridoidic compounds (PI) has a strong antitumor effect on THP-1 cells, which is demonstrated by the significant decrease of cell viability depending on the increase in concentration and by the amplification of apoptosis and necrosis processes leading to tumor cell death.

The increase in the necrosis level of tumor cells comparatively with apoptosis level suggests that the level of the PI concentration used in in vitro experiments is too high for this type of cell line.

Prebiotic activities of the polysaccharides fraction are due to extracellular secretion of hydrolases of lactobacilli, which in this way are able to metabolize galactose, sucrose, and arabinoxylan taking into account that the L-arabinose, D-galactose, and D-galacturonic acid are the main components in crude polysaccharides fractions [53,54,55].

The different behavior of *S. cerevisiae* can be explained by the fact that the yeast is not able to metabolize the main components of the polysaccharidic fraction from Plantain (like arabinose regarding galactose, *S. cerevisiae* can use this substrate, but with much less yield in comparison with dextrose [56,57].

## 5. Conclusions

In this study, three types of extracts obtained from leaves of *P. lanceolata*, which contain polysaccharides (PP), flavonoids (PF), and iridoids (PI), were studied in terms of biological activities of high interest to the pharmaceutical industry.

The antioxidant activity of the PF fraction and the HPTLC results represent proof that PF contains flavonoids such as luteolin, rutin (luteolin-7-O-glucoside), kaempferol, and cymaroside, as well as the hydroxycinnamic acids (caffeic acid). In terms of prooxidant activities, we supposed that the fractions PP and PI do not possess polyphenolic compounds in a sufficient concentration to act as radical scavengers, and most probably this is a reason for which these two fractions isolated from aqueous media act as prooxidants.

The main conclusions and potential applicability were as follows: the three extracts were studied (PP, PI, PF), and all indicated moderate antifungal activity against dermatophytes such as *M. canis*, *T. mentagropythes*, *M. gypseum*, and *S. brevicaulis* and micromycetes type *Penicillium* sp. and *Aspergillus* sp.; in vitro studies on the leukemic cell line type THP-1 indicated moderate to augmented antitumor activity, the PF and PI fractions suggesting an inhibitory mechanism based on the stimulation of the apoptosis process. Specifically, the antitumor activity for the three bioproducts decreased in the order PI > PF > PP; the PP extract confirmed the prebiotic potential of some important microorganisms found in human microbiota, in particular on *L. plantarum*, *L. reuteri*, and *L. casei*. In conclusion, in vitro experiments revealed the antitumor effect of the analyzed fractions obtained from *P. lanceolata*, which encourages the extension of studies on several types of normal cells and tumor cells.

## Figures and Tables

**Table 1:** Interplanar distances obtained for the sample PI.

Peak	2θ (°)	θ (°)	d, Å	Peak Intensity
1	4.8883	2.4442	18.0626	456.9
2	25.5078	12.7539	3.4892	51.02
3	27.2186	13.6093	3.2736	79.41
4	27.9222	13.9611	3.1927	321.18
5	28.3559	14.1780	3.1449	21501.72
6	40.5385	20.2693	2.2235	14142.32
7	47.7775	23.8888	1.9021	40.15
8	50.1652	25.0826	1.8170	162.86
9	58.5917	29.2959	1.5742	2821.06

## Abbreviations

MRS	cultivation media for Lactobacillus species
YPG	cultivation media that contain yeast extract, peptone, and glucose
DPPH	2,2-diphenyl-1-picrylhydrazyl (i.e., 2,2-Diphenyl-1-(2,4,6-trinitrophenyl)hydrazin-1-yl)
GAE	Galic Acid equivalents
MRC-5	diploid cell culture line composed of fibroblasts
HeLa	cervical cancer cell line
MCF-7	human breast cancer cell line
HT-9	colorectal tumor cell line
HL-60	promyelocytic leukemia cell line
K562	human chronic myeloid leukemia cell line
CCRF-CEM	acute lymphoblastic leukemia cell line
P3HRI	Burkitt lymphoma cells
P338	murine leukemia cell line
Bax	proapoptotic protein; apoptosis regulator
Bcl-2	proapoptotic protein apoptosis regulator
p53	tumor suppressor gene
p21	protein which regulates cell proliferation by inhibiting the cell cycle through the cyclin kinase pathway
G1	cell phase in which the cell grows physically larger copies of organelles and makes the molecular building blocks it will need in later steps
G0	cell phase also known as the resting phase is the phase of the cell cycle during which a cell is neither dividing nor preparing to divide
